# Beyond genome-wide scan: Association of a cis-regulatory *NCR3* variant with mild malaria in a population living in the Republic of Congo

**DOI:** 10.1371/journal.pone.0187818

**Published:** 2017-11-09

**Authors:** Sabrina Baaklini, Sarwat Afridi, Thy Ngoc Nguyen, Felix Koukouikila-Koussounda, Mathieu Ndounga, Jean Imbert, Magali Torres, Lydie Pradel, Francine Ntoumi, Pascal Rihet

**Affiliations:** 1 INSERM, UMR1090 TAGC, Marseille, France; 2 Aix-Marseille Université, Marseille, France; 3 Fondation Congolaise pour la Recherche Médicale (FCRM), Villa D6, Cité OMS AFRO, Brazzaville, République du Congo; 4 Faculté des Sciences et Techniques, Université Marien Ngouabi, Brazzaville, République du Congo; 5 Unité de Recherche sur le Paludisme, Centre d’Etudes sur les Ressources Végétales (CERVE), Brazzaville, République du Congo; 6 Institute for Tropical Medicine, University of Tübingen, Tübingen, Germany; Universidade de Sao Paulo Instituto de Ciencias Biomedicas, BRAZIL

## Abstract

Linkage studies have revealed a linkage of mild malaria to chromosome 6p21 that contains the *NCR3* gene encoding a natural killer cell receptor, whereas *NCR3-412G>C* (rs2736191) located in its promoter region was found to be associated with malaria in Burkina Faso. Here we confirmed the association of rs2736191 with mild malaria in a Congolese cohort and investigated its potential cis-regulatory effect. Luciferase assay results indicated that rs2736191-G allele had a significantly increased promoter activity compared to rs2736191-C allele. Furthermore, EMSAs demonstrated an altered binding of two nuclear protein complexes to the rs2736191-C allele in comparison to rs2736191-G allele. Finally, after *in silico* identification of transcription factor candidates, pull-down western blot experiments confirmed that both STAT4 and RUNX3 bind the region encompassing rs2736191 with a higher affinity for the G allele. To our knowledge, this is the first report that explored the functional role of rs2736191. These results support the hypothesis that genetic variation within natural killer cell receptors alters malaria resistance in humans.

## Introduction

Malaria caused by *Plasmodium falciparum* parasites remains a major global public health problem with 212 million new cases and 429 000 deaths in 2015 according to the WHO [1]. Sub-Saharan Africa accounted for most of these new cases and deaths (nearly 90%).

The contribution of host genetic variants to susceptibility to *Plasmodium falciparum* malaria has been widely studied since the discovery of the protective role of sickle cell mutation against malaria [2–4]. Indeed, malaria parasites have exerted strong selective pressure on the human genome in endemic areas, leading to the progressive accumulation of genetic adaptations in these populations [5]. Linkage and association studies have reported several associations between polymorphisms of candidate loci and various malaria-related phenotypes [6]. These phenotypes, which characterize malaria severity, include high parasitaemia, fever, anaemia and cerebral malaria.

Several linkage analyses conducted in different African ethnic groups provided evidence of linkage between the 6p21 locus and mild malaria [7–9]. In addition, several polymorphisms found under the linkage peak, and more precisely, within *TNF*, *LTA* and *NCR3* genes, were independently associated with subphenotypes of mild malaria such as parasitaemia or mild malaria attack in Burkina Faso [10–12]. Among those, several *TNF* and *LTA* polymorphisms have been characterized as cis-regulatory polymorphisms [13–18]. However, the functional effect of the polymorphism located within the promoter of *NCR3* gene (*NCR3-412G>C*, rs2736191), which encodes NKp30 receptor, was unknown.

The natural cytotoxicity receptor NKp30 (natural cytotoxicity triggering receptor 3, *NCR3*) has been identified to trigger NK-cell–mediated killing of several tumor cell lines [19], virus-infected cells [20], fungal cells [21], and also dendritic cells [22,23]. In patients with acute myeloid leukaemia, down-regulation of NKp30 expression has been shown to correlate with a decreased NK cells cytotoxicity [24]. Several studies provided evidences that NK cells directly and specifically interact with infected erythrocytes [25–27] resulting in their killing through cytotoxic activity [28–32] and this lysis may be triggered by the recognition of ligands expressed on parasitized RBC surface by NK cell receptors, among them NKp30 receptor [30,33]. Besides, NKp30 engagement at the NK cell surface can lead to the production of IFNγ, which has been shown to correlate with a reduced risk of mild malaria [34,35]. Therefore, polymorphisms that reduce NKp30 expression may increase the risk of mild malaria. Also, we hypothesized that the rs2736191-C allele associated with mild malaria in Burkina Faso binds less efficiently transcription factors recruited to the promoter, and that it diminishes the expression of its target gene. Here we investigated the cis-regulatory effect of rs2736191, and its association with mild malaria in a population living in the Republic of Congo, where there are limited human genetic data in relation with infectious diseases [36].

## Materials and methods

### Subjects and phenotypes

The study subjects have resided in Kinsana, Mbouono and Ngoko, three suburban areas close to each other and located in Makélékélé, one of the seven districts of Brazzaville (Congo) [37]. Three hundred and seventeen individuals were recruited between April and June 2010 and followed up during one year. Blood samples were collected at Makélékélé health division, which is located at 6km from the study area in the centre of the district; a written informed consent signed by the parents or legal guardians was obtained for the children. In this area, malaria is transmitted throughout the year with *P*. *falciparum* being the predominant species and *Anopheles gambiae* the main mosquito vector [38,39]. All the recruited individuals belonged to Bantu ethnicity, and were aged from one to nine years. Age mean was 4.34 (SD = 2.55), and sex ratio was 1.13 (167 Male and 148 Female). During the study period, if a child presented any symptoms related to clinical malaria, he was examined at the health center and treated in case of malaria episode (positive thick, thin blood smears and axillary temperature ≥37.5°C). Thick and thin blood smears were independently read by at least two trained biologists. The slides were read using an oil immersion lens at a 100x magnification. A slide was considered negative when no asexual parasite was observed after reading at least 100 thick fields. For positive results, asexual parasites were counted against at least 200 leucocytes and expressed as the number of asexual parasites per μl, assuming 8,000 leucocytes/μl of blood. The children who experienced at least one clinical malaria attack were classified in a group as mild malaria, whereas the children without any reported mild malaria episode in spite of the carriage of parasites during the follow-up period were considered as asymptomatic, as and were classified in a group as asymptomatic children. This led to determine a binary mild malaria phenotype. In addition, the number of malaria episodes was registered, and was considered as another phenotype. The ethical approval was given by the Institutional Ethics Committee for Research on Health Sciences of the Republic of Congo.

### DNA extraction and rs2736191 genotyping

Genomic DNA was extracted from 200 μl of peripheral whole blood samples from patients and asymptomatic individuals using QIAamp DNA Blood Mini Kit (Qiagen, Hilden, Germany); DNA samples were then amplified by Illustra GenomiPhi V2 DNA Amplification kit (GE Healthcare Life Sciences, Velizy-Villacoublay, France) following the manufacturer’s instructions. PCR amplification of DNA fragments containing rs2736191 with forward (5’-GATGGGTCTGGGTACTGGTG-3’) and reverse (5’-GGGATCTGAGCAGTGAGGTC-3’) primers was described by Delahaye *et al*. [10]. For RFLP analysis, the PCR product of rs2736191 was digested with 10 units of PfoI restriction endonuclease (Thermo Fisher Scientific, Waltham, MA, USA) at 37°C overnight. The digestion products were loaded on to Agilent DNA 1000 chips and analyzed on the 2100 Bioanalyzer, per manufacturer's instructions. Validation of the PCR-RFLP method was assessed by sequencing 37 PCR products of rs2736191 (corresponding to 37 individuals) on a Beckman coulter CEQ 8800 sequencer according to manufacturer’s protocol (Beckman Coulter, Brea, CA, USA). Three hundred and fifteen DNA samples of the 317 samples were successfully genotyped (S1 Table).

### Statistical analysis

Hardy-Weinberg equilibrium was tested and allelic frequencies were calculated, as described [40]. To estimate the association of rs2736191 polymorphism with the binary mild malaria phenotype, we used a chi-square test. We further performed a logistic regression analysis to evaluate the odds ratios (OR) and their 95% confidence intervals (CIs). Moreover, we assessed the association of the investigated SNP with the number of episodes by a Kruskal and Goodman’s gamma test and the Poisson regression method. To assess the effect of the SNP on the promoter activity, Student’s t-test was used after checking the normality of the distributions by using the Shapiro-Wilk method and their variance equality by using a Fisher test. All analyses were performed by using either R or SPSS software (SPSS, Boulogne, France). Only terms significant at the 5% level were retained.

### *NCR3* linkage disequilibrium analyzes

To evaluate the linkage disequilibrium status of the region where rs2736191 is located, we explored the 1000 Genome Project data [41] for the Yoruba population through Ensembl browser [42].

### Cell culture

Human NK92 cells (ATCC, Manassas, VA, USA, ATCC® CRL-2407™) were cultured in alpha MEM (Thermo Fisher Scientific, Waltham, MA, USA), supplemented with 12.5% horse serum, 12.5% fetal calf serum (FCS), 2 mM L-glutamine, 1.5 g/l sodium bicarbonate, 0.2 mM inositol, 0.1 mM 2-mercaptoethanol, 0.02 mM folic acid and 100 U/ml recombinant IL-2 (PeproTech Inc., Rocky Hill, NJ, USA). K-562 cells (ATCC® CLL-243™) were grown in Gibco® RPMI 1640 medium (Thermo Fisher Scientific, Waltham, MA, USA) supplemented with 20% fetal bovine serum.

### Luciferase assay

A 663bp DNA fragment upstream the *NCR3* translation start site (chromosome 6: 31592727–31593389 according to the hg38 assembly) was cloned by “Sticky end PCR Cloning” method. Briefly, two PCR were performed on human gDNA from NK92 cell line using Herculase II Fusion enzyme (Agilent Technologies, Santa Clara, CA, USA, cat# 600679): PCR1 with the forward primer (CGCGTACCCAACCAAACACA) and the reverse primer (GAAGATGTCCCAGTTGGCGA), and PCR2 with the forward primer (TACCCAACCAAACACACACA) and the reverse primer (TCGAGAAGATGTCCCAGTTG). The PCR products from PCR1 and PCR2 were mixed and hybridized together to create a 663pb DNA fragment with XhoI and MluI sticky end. This fragment was cloned into the MluI-XhoI sites of pGL3-Enhancer Vector (Promega, Madison, WI, USA, cat# E1771,), which contained the firefly luciferase coding sequence. Initially, the pGL3 construct obtained, presented the G allele of rs2736191. To obtain the pGL3 construct containing the rs2736191-C allele, site-directed mutagenesis using the Q5 Site-Directed Mutagenesis Kit (New England Biolabs, Ipswich, MA, USA, cat#E0554S) was performed with primers designed by NEBaseChanger tool provided by the supplier (forward: AGGGCTCCTGcAGGCTTGTTC, reverse: CCTTGGCCCAGAAGCTAAC, annealing temperature: 67°C). K562 transfection was performed with Neon™ Transfection System (Invitrogen) following constructor instructions. For each out of the 6 tests performed, 10^6^ cells were co-transfected with 1 μg of control vector (empty pGL3-Enhancer vector) or with 1μg of tested construction (pGL3-Enhancer vector containing the rs2736191-G (p-G) or rs2736191-C (p-C) *NCR3* promoter) and 200ng of pRL-SV40 (a plasmid encoding Renilla luciferase from Promega), which was used as a transfection efficiency control. Transfected cells were maintained at 37°C in 5% CO2 during 24h. Firefly and Renilla luciferase values were obtained by analyzing 20μl of cell lysate according to standard instructions provided in the Dual Luciferase Kit (Promega cat# E1910) in a TriStar LB 941 Multimode Microplate Reader (Berthold technologies, Thermo Fisher Scientific, Waltham, MA, USA). The firefly luciferase activity of each sample was normalized by Renilla luciferase activity and adjusted by pGL3-enhancer mean activity.

### Electrophoretic mobility shift assay

NK92 nuclear extracts were prepared with the NE-PER Nuclear and Cytoplasmic Extraction Reagents (Thermo Fisher Scientific, Thermo Fisher Scientific, Waltham, MA, USA). Oligonucleotides were designed based on the genomic sequence surrounding rs2736191; the SNP position is shown in bold: 5’-biotin- GGAGGGCTCCTG**G****/****C**AGGCTTGTTCTGG. All oligonucleotides were ordered from Eurofins (Eurofins, Strasbourg, France). We performed gel mobility shift assays with the LightShift Chemiluminescent EMSA Kit (Thermo Fisher Scientific) according to the manufacturer’s protocol. For competition assays, 200 or 400-fold excess unlabelled double-stranded variant oligonucleotides were incubated with the extracts (23°C, 30 min) before probe addition. Bound complexes were separated on 6% polyacrylamide gels and blotted onto membrane. The blot was processed with a streptavidin-horseradish peroxidase conjugate-based detection method.

### In-silico identification of transcription factors candidates

An in silico prediction of transcription factor (TFs) candidates was performed using ReMap [43] and ENCODE catalogs of ChIP-seq peaks in lymphocyte cells. To detect a co-localization between these transcription factors binding sites and SNPs, we visualized the TFs ChIP-seq peaks in the UCSC Genome browser [44]. Then, to select TFs candidates, for which the peak of ChIP-seq co-localized with the SNP of interest we retained those with the maximum signal represented in black in the UCSC genome browser for the ENCODE catalog [45]. In the case of ReMap catalogs (2013 unpublished and [43]), we retained all the TFs, for which the peak of ChIP-seq co-localized with the SNP.

### DNA pulldown and Western blot assays

DNA pulldown assay was performed as described previously [46]. Briefly, complementary TEG-biotinylated oligonucleotides encompassing the rs2736191-G/C binding site, GGAGGGCTCCTG**G**/**C**AGGCTTGTTCTGG (the critical SNP is in bold), were annealed to form dsDNA. TEG-biotinylated dsDNA (2 μg) was conjugated to 100 μl streptavidin-bound magnetic beads (Thermo Fisher Scientific, Thermo Fisher Scientific, Waltham, MA, USA, Dynabeads, M280; *Dynal*) in binding/washing buffer (10 mM Tris–HCl [pH 8], 1 mM EDTA, and 0.1 M NaCl) for 30 min at room temperature. Conjugated DNA was collected with a magnetic particle concentrator. DNA-conjugated beads were then blocked with 0.5% BSA in TGEDN buffer (120 mM Tris–HCl [pH 8], 1 mM EDTA, 0.1 M NaCl, 1 mM DTT, 0.1% Triton X-100, and 10% glycerol) at room temperature for 1 h. Beads were washed in TGEDN buffer and resuspended in 50 μl TGEDN. Ten-microliter beads conjugated to 2 μg DNA were equilibrated with TGEDN buffer and incubated with 500 μg NK92 nuclear extracts and 20 μg herring sperm DNA (Sigma-Aldrich, Saint-Louis, MO, USA) at 4°C overnight. Beads were washed in TGEDN buffer, and bound materials were eluted in 20 μl of the same buffer supplemented with 0.5% SDS and 1 M NaCl.

The eluted proteins were separated by 10% SDS-polyacrylamide gel and transferred electrophoretically to a PVDF membrane. The membrane was then blocked in a 5% nonfat dry milk/Tris-buffered solution and incubated with primary antibody overnight. For STAT4, STAT5A, STAT5B detection a 1:250 dilution of an anti-STAT4 rabbit polyclonal antibody (sc-489, Santa Cruz Biotechnology Inc., Dallas, Texas, USA), anti-STAT5A rabbit polyclonal antibody (sc-1081, Santa Cruz Biotechnology Inc, Dallas, Texas, USA), or an anti-STAT5B mouse monoclonal antibody (sc-1656, Santa Cruz Biotechnology Inc.) was used. For STAT3 and EGR1 detection, a 1:500 dilution of an anti-STAT3 mouse monoclonal antibody (sc-8059, Santa Cruz Biotechnology Inc.) or an anti-EGR1 rabbit polyclonal antibody (sc-189, Santa Cruz Biotechnology Inc.) was used. And for RUNX3 detection a 1:1000 dilution of an anti-RUNX3 mouse monoclonal antibody was used (ab40278, Abcam, Cambridge, UK). The unbound primary antibody was removed by washing the membrane with 0.1% Tween/Tris-buffered solution, followed by incubation with horseradish peroxidase-conjugated secondary antibody diluted at 1:10000 in 0.5% nonfat dry milk/Tween/Tris-buffered solution. Proteins were visualized using ECL (GE Healthcare Life Science, Velizy-Villacoublay, France).

## Results

### Association of rs2736191 with mild malaria and the number of episodes

Three hundred and fifteen individuals were successfully genotyped. The observed genotype frequencies conformed to the Hardy-Weinberg equilibrium (P = 0.5086). The allelic frequencies for the rs2736191-G and rs2736191-C alleles were 0.73 and 0.27, respectively.

To assess the association of rs2736191 polymorphism with mild malaria in the study population, we performed a Chi-square test with 1df and a logistic regression analysis. We further evaluated the odds ratios and their 95% confidence intervals. Among the 315 individuals genotyped, 176 presented at least one malaria attack during the study (Table 1). We found that carriers of the rs2736191-C allele had a significantly higher risk of mild malaria attack without age as a covariate (P = 0.033), and with age as a covariate (P = 0,029) (Table 2). We further looked at the age distribution in the study population. Fifty-three % of the individuals were under 5 years old (Table 3). Thus, we splitted the population into two groups (subjects under 5 years and subjects over 5 years), and tested an interaction between Age and rs2736191. There was a significant interaction (P = 0.001; OR = 2.80, 95% confidence interval 1.53–5.12), indicating that subjects under 5 years and subjects over 5 years differed in the effect of rs2736191. Thus, we conducted an analysis after stratifying individuals by age categories. For the over-5 sub-population, there was a significant association between the rs2736191-C allele and mild malaria attack with a better p-value (P = 0.008) (Table 2), whereas there was no longer an association for the under-5 sub-population.

**Table 1:** Clinical data of individuals for rs2736191 polymorphism.

Genotype	Unaffected Individuals	Affected Individuals	Total
GG	83 (61.1%)	85 (48%)	168 (53.3%)
GC	43 (31.2%)	78 (44%)	121 (38.4%)
CC	12 (8.7%)	14 (8%)	26 (8.3%)
Total	138 (100%)	177 (100%)	315 (100%)

**Table 2:** Association tests of rs2736191 with mild malaria.

	Whole study population			Individuals < 5^a^			Individuals ≥ 5^b^		
	*P*	OR	95%CI	*P*	OR	95%CI	*P*	OR	95%CI
Univariate anlysis^c^	0.033	1.63	1.04–2.56	0.59	1.18	0.65–2.16	0.008	2.59	1.28–5.25
Multivariate analysis^d^	0.029	1.67	1.05–2.63	0.52	1.22	0.66–2.27	0.009	2.57	1.27–5.20

^a^ Children under 5 years of age

^b^ Children over 5 years of age

^c^ Assuming a dominant model (GG vs. GC+CC), p-values and Odd ratios were calculated according to logistic regression test for the affected/unaffected phenotype without age as a covariate

^d^ Assuming a dominant model (GG vs. GC+CC), p-values and Odd ratios were calculated according to logistic regression test for the affected/unaffected phenotype; the logistic model included age as a covariate

**Table 3:** Repartition of rs2736191 polymorphism in the study population according to age.

Genotype	Study population		
	Age < 5	Age ≥ 5	Total
GG	89 (52.7%)	79 (54.1%)	168 (53.3%)
GC	63 (37.3%)	58 (39.7%)	121 (38.41%)
CC	17 (10%)	9 (6.2%)	26 (8.3%)
Total	169 (100%)	146 (100%)	315 (100%)

Furthermore, we tested the correlation between rs2736191 genotype and the number of mild malaria episodes (Tables 4, and S2 and S3 Tables) using Kruskal and Goodman’s gamma test. We found that rs2736191-C allele was significantly associated with an increased number of episodes (P = 0.036). In addition, we used the Poisson regression to assess the correlation between rs2736191 genotype and the number of mild malaria episodes, after taking into account age. The association was no longer significant (P = 0.087). We also performed an analysis after stratifying by age categories (<5 years and ≥5 years) and detected a significant association between the rs2736191-C carriage and an increased number of episode in the over-5 sub-population (P = 0.006), whereas there was no longer an association for the under-5 sub-population (P = 0,69). We confirmed the association of rs2736191 with the number of mild malaria episodes for the over-5 sub-population by using the Poisson regression when taking into account age (P = 0.020).

**Table 4:** Proportion of mild malaria episodes according to rs2736191 polymorphism.

Genotype	Number of mild malaria episodes						Total
	0	1	2	3	4	5	
GG	83 (60.1%)	54 (49.6%)	16 (43.2%)	6 (37.5%)	7 (53.8%)	2 (100%)	168 (53.3%)
GC	43 (31.2%)	47 (43.1%)	21 (56.8%)	6 (37.5%)	4 (30.8%)	0 (0%)	121 (38.4%)
CC	12 (8.7%)	8 (7.3%)	0 (0%)	4 (25%)	2 (15.4%)	0 (0%)	26 (8.3%)
Total	138 (100%)	109 (100%)	37 (100%)	16 (100%)	13 (100%)	2 (100%)	315 (100%)

### rs2736191 affects *NCR3* expression

**Fig 1:**
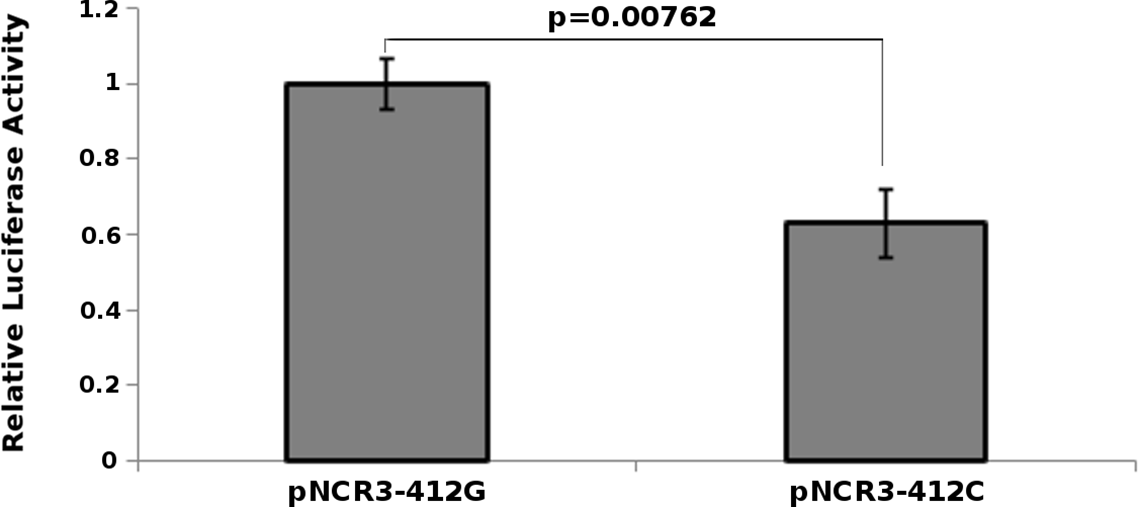
rs2736191 polymorphism affects *NCR3* expression. Luciferase reporter gene assays with constructs containing the rs2736191-G (p-G) or rs2736191-C (p-C) *NCR3* promoter in K-562 cell line. All constructs were co-transfected with pRL-SV40 to standardize transfection efficiency and luciferase activity for each sample was adjusted by the empty pGL3-Enhancer. Fold increase was measured by defining the activity of p-G vector as 1. Data shown are the means ± SE from 2 independent transfection experiments, each performed in triplicate. The rs2736191-G-containing *NCR3* promoter drove significantly higher reporter gene expression (~37%) than the rs2736191C-containing *NCR3* promoter (P < 0.05, as shown in the Figure). Statistical analysis was performed using two-tailed Student’s t-test after controlling the normality of the data and the equality of their variance.

To examine whether the rs2736191 G>C affects *NCR3* promoter activity; we performed six gene reporter assays in K-562 cells. As shown in Fig 1, the transcriptional activity driven by the p-C construct containing the rs2736191-C allele decreased nearby 37% compared with that driven by the p-G construct containing the rs2736191-G allele (P = 0.00762).

### Effect of rs2736191 polymorphism on transcription factors binding to the promoter

**Fig 2:**
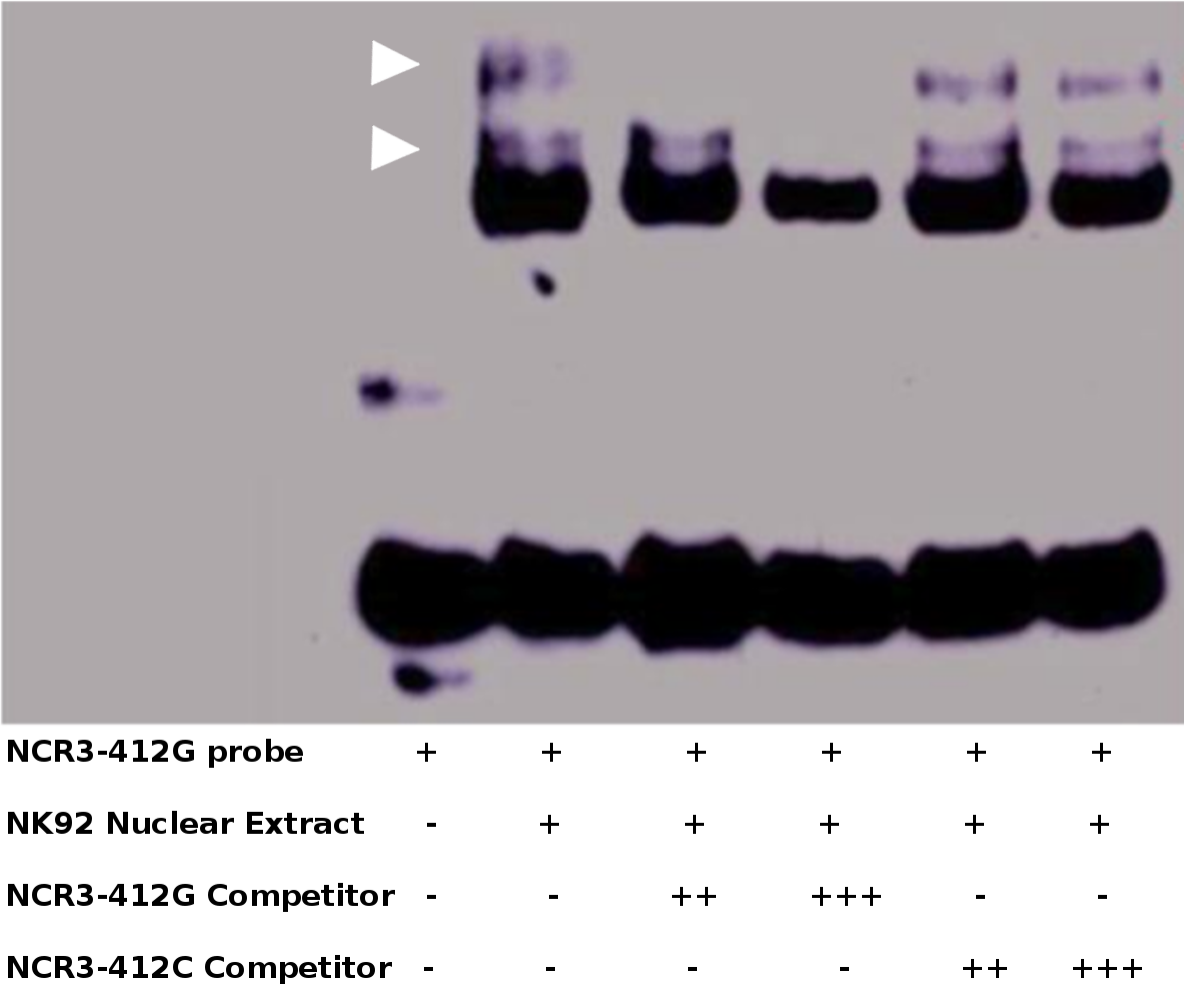
Effect of rs2736191 polymorphism on transcription factors binding. EMSA with biotin-labelled probes containing the G allele of rs2736191 and incubated with NK92 nuclear extracts. Competition was performed with 200x (lane 3 and 5) or 400x (lane 4 and 6) excess of double-stranded DNA containing either the G or the C variants. Two arrows indicate two specific nuclear proteins that interact with rs2736191-G probe. The G variant was more effective in competing these two nuclear proteins compared to the C variant.

The cis-regulatory effect of rs2736191 polymorphism on transcription factors binding was tested through an EMSA. Two major DNA-protein complexes were revealed, as shown in Fig 2. To assess if these 2 complexes bind specifically and preferentially one of the two variants, we performed competition assay with an increasing amount of non-labelled probes containing either the C or the G allele. Both complexes appeared to have a stronger affinity for the G allele as only the rs2736191-G unlabelled probe abolished very efficiently the binding of these complexes compared to the rs2736191-C unlabelled probe (Fig 2).

### STAT4 and RUNX3 bindings are altered by rs2736191 polymorphism

An in silico prediction of transcription factor candidates using ReMap and Encode catalogs of ChIP-seq peaks in lymphocyte cells was performed and visualized in the UCSC Genome browser. We explore the co-localization of ChIP-seq peaks with rs2736191 and the SNPs in strong linkage disequilibrium with it. The SNP exhibiting the strongest linkage disequilibrium with rs2736191 was rs2256974 (r^2^ = 0.699). Rs2256974 is located in a non-conserved region with neither DNAse1 footprinting nor H3K27Ac mark co-localization and without evidence of transcription factors binding site, in contrast to rs2736191 polymorphism. Indeed, rs2736191 was co-localized with STATs family members (ReMap catalog) and SP1, RUNX3, EGR1, EBF1 (ENCODE catalog) transcription factors. As SP1 and EBF1 signals were quite low, we selected STATs family members, RUNX3 and EGR1 for further in vitro analysis.

Thus, we pulled-down NK92 nuclear proteins that bound a 26 bp double-stranded biotinylated probe containing either the rs2736191-C or rs2736191-G variant using streptavidin-magnetic beads. We further analyzed the bound proteins by performing a western-blot with antibodies directed against STATs, RUNX3 and EGR1.

**Fig 3:**
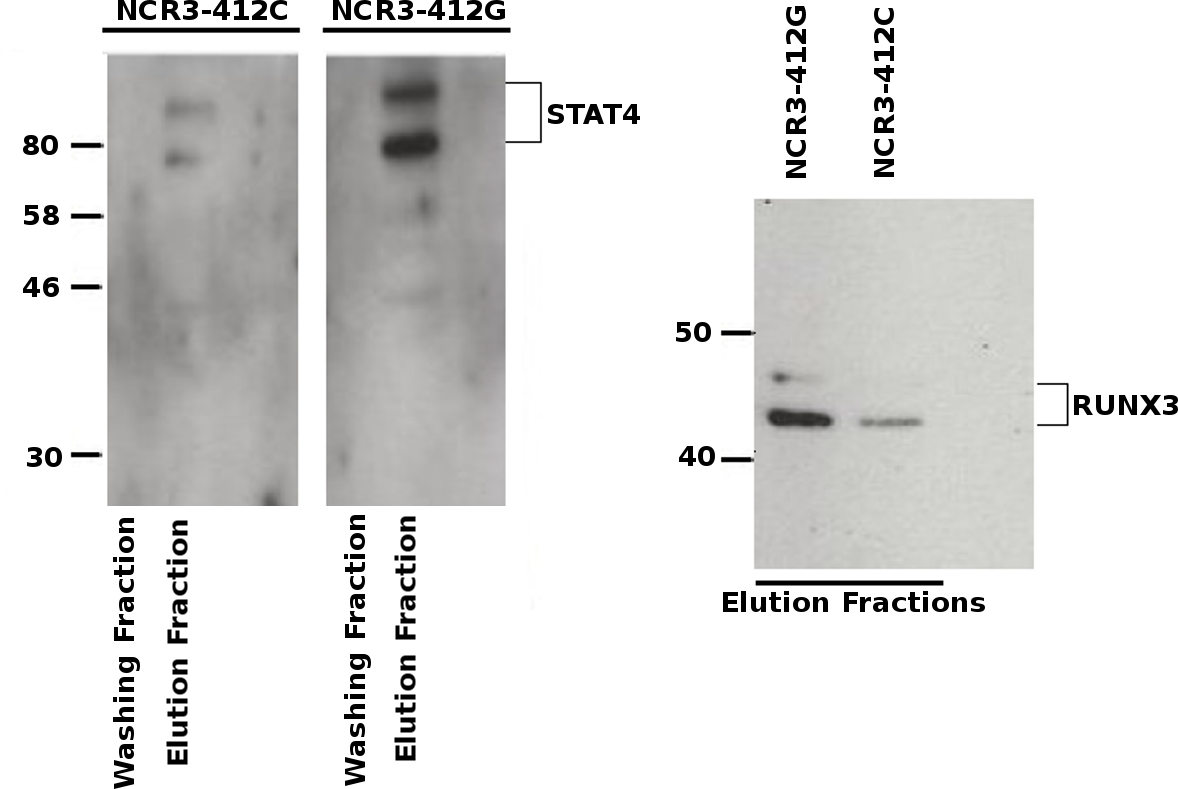
Differential binding of transcription factor candidates to rs2736191 polymorphism. DNA pull-down assay was performed with nuclear extracts prepared from NK92 cells and complementary biotinylated oligonucleotides encompassing the rs2736191-G/C binding sites. After the incubation, the nuclear protein bound were isolated using magnetic beads coupled with streptavidin and subjected to 12% SDS-PAGE and Western blotting for the transcription factors STAT4 and RUNX3. The strongest intensity obtained with the G probe, for both STAT4 and RUNX3, suggests a preferential binding of these transcription factors in presence of this allele compared to the rs2736191-C allele.

We did not observe a binding of STAT3, STAT5A, STAT5B and EGR1 transcription factors to the sequence containing the rs2736191 polymorphism (data not shown). In contrast, we evidenced that RUNX3 and STAT4 bound the sequence containing the rs2736191 polymorphism, and that the binding to the one presenting the G allele was stronger when compared to the one presenting the C allele (Fig 3).

## Discussion

We have previously evidenced a genome-wide significant linkage of mild malaria with chromosome 6p21, which contains *NCR3* encoding for the Nkp30 receptor [7], and detected a genetic association between rs2736191 polymorphism (located in *NCR3* promoter region) and mild malaria in the same Burkinabe population [10]. The location of the SNP within the promoter suggested that this SNP is a *cis*-regulatory variant controlling *NCR3* expression. In this study, we thus evaluated the association between rs2736191 and mild malaria in a Congolese cohort and assessed its cis-regulatory effect.

We found that rs2736191-C carriers had a significant increased number of mild malaria episodes compared to the non-carriers. In the case of the affected vs. unaffected phenotype, the multivariate logistic regression analysis revealed that, as well as the study in Burkina Faso [10], rs2736191 polymorphism was significantly associated with the uncomplicated form of malaria. It should be stressed, nevertheless, that the present study in Congo was based on a passive follow up, whereas the study in Burkina Faso was based on an active follow-up; also, the passive follow-up can be considered a limit of the present study. In spite of this limit, the odds ratio obtained for the over-5 population was very close to the odd-ratio obtained in the Burkinabe population (OR = 2.59 and OR = 2.93, respectively), which was mainly composed of children over-5 years of age.

Luciferase assays performed in K-562 cells expressing NCR3 mRNA [47] further supported a functional role of this SNP. Indeed, we observed that the p-G construct presenting rs2736191-G allele had a 37% increased promoter activity compared to the p-C construct presenting rs2736191-C allele. Furthermore, gel shift experiments, performed with NK92 nuclear protein extracts, showed an increased binding affinity of the rs2736191-G allele to two nuclear protein complexes compared to the rs2736191-C allele.

On the basis of bioinformatic analyses, we identified several transcription factor candidates corresponding to these complexes. Furthermore, pull-down western-blot experiment confirmed that both STAT4 and RUNX3 bound the region encompassing rs2736191 with a higher affinity for the G allele. STAT4 is essential for IL-12 mediated cytotoxicity and IFNγ production in mouse and human NK cells [48] and a direct binding to perforin promoter has already been reported in human NK cells [49]. Concerning RUNX3 transcription factor, it is highly expressed in natural killer cells [50] but few is known about its function in these cells. Nevertheless, Levanon *et al*. have shown that, in mice, RUNX3 cooperation with ETS and T-box transcription factors regulates the interleukin-15-mediated transcription program during activation of Natural Killer cells [51]. Most of the time, the three NCR1, NCR2, and NCR3 were shown to be co-expressed, suggesting a co-regulation of the three NCRs, whereas a reduced expression of the three NCRs has been associated with impaired NK cytolytic function [52,53]. Moreover, RUNX3 has been shown to also regulate NCR1 expression [54] and ETS-1 transcription factor was also shown to bind to a specific and common region in the NCRs promoters [55]. Our results further support that RUNX3 can be one of these key regulatory factors that sustain the coordinated regulation of three NCRs.

The role of NK cells in malaria has been extensively investigated evoking either their cytokine production function [27,56,57] or their cytotoxic response to infected red blood cells [28–33]. According to Mavoungou *et al*. [30,33], the mechanism underlying NK cells cytotoxicity in *Plasmodium falciparum* malaria, is the recognition of the DBL1α domain of the parasitic antigen PfEMP1 by the NKp30 activating receptor, resulting in parasitized red blood cells lysis. All together these results support the biological model involving NKp30 receptor in the cytotoxic response to parasitized RBC. However, NK cell receptors and infected RBC ligands leading to this cytotoxic response remains controversial. Indeed, PfEMP1 that is known to bind several other molecules such as ICAM-1, complement receptor 1 (CR1), heparan sulfate (HS), chondroitin sulfate A (CSA) and CD36 [58], have failed to activate NK cell cytotoxicity in other studies [26,59]. Nevertheless, in the first study the authors tested the interaction between PfEMP1 and CSA, while in the second study, authors have looked for NK cytotoxicity markers after 6 hours of co-culture with either infected red blood cells (iRBC) expressing PfEMP1 or iRBC not expressing PfEMP1 arguing that the ligand density of native PfEMP1 on the surface of iRBC is not enough to activate NK cells cytotoxicity compared to *in vitro* high concentration of peptidic DBL-1α as proposed by Mavoungou *et al*. [33]. Though, because of its binding abilities with various receptors, its diversity and its variable density on iRBC [60,61], involvement of PfEMP1 and its interactors on NK cells surface merits further investigation.

Besides, other ligands and receptors have been proposed to be involved in the cytotoxic response of NK cells, among them, Hsp70, which is a “self” stress ligand [62] or LFA-1, which is a molecule essential for stable contact with target cells [29,63]. As triggering of at least two activating receptors is essential for efficient NK cells cytotoxicity and cytokine release [64], a synergistic effect also suggested by Mavoungou *et al*. [33] in a malarial context, these results do not seems contradictory. However, this synergistic activation requires further investigation.

In conclusion, we replicated and extended the association of rs2736191 with mild malaria in an independent population living in Central Africa. Moreover, we evidenced its effect on the promoter activity and on the binding of nuclear proteins, including transcription factors, such as STAT4 and RUNX3. These results support the hypothesis that rs2736191 alters the activation of NK cells, and thus influences human malaria resistance.

## Supporting information

S1 Table Phenotype and genotype data set. (PDF)

S2 Table Proportion of mild malaria episodes according to rs2736191 for children under 5 years. (PDF)

S3 Table Proportion of mild malaria episodes according to rs2736191 for children over 5 years. (PDF)
