# Supplementary material for: Beyond genome-wide scan: Association of a cis-regulatory NCR3 variant with mild malaria in a population living in the Republic of Congo
Source: PLoS One. 2017 Nov 9;12(11):e0187818. doi: 10.1371/journal.pone.0187818 (PMC5679660; doi:10.1371/journal.pone.0187818)
Supplement: S1 Table — (PDF) [file pone.0187818.s001.pdf]

S1 Table. Phenotype and genotype data

Patients	Age (years)	rs2736191	Mild Malaria	Number of mild malaria episodes
1	9	GC	0	0
2	9	GG	1	1
3	4	GC	0	0
4	8	CC	1	4
5	3	GC	1	4
6	8	GC	1	1
7	4	GC	1	1
8	8	GG	1	5
9	2	CC	1	1
10	4	GG	1	3
11	3	GG	1	2
12	8	CC	1	1
13	2	GG	0	0
14	4	GG	1	1
15	4	GG	1	1
16	7	GG	0	0
17	6	GG	0	0
18	7	GG	1	4
19	7	GG	1	1
20	1	GC	0	0
21	2	GC	1	1
22	5	GC	1	2
23	3	GC	1	1
24	6	GG	1	1
25	7	GC	1	1
26	8	GG	0	0
27	6	GG	1	1
28	1	GG	1	1
29	6	GC	1	1
30	7	GG	1	2
31	7	GC	1	2
32	3	GC	1	2
33	5	GG	0	0
34	4	GC	1	1
35	6	GG	0	0
36	3	GG	0	0
37	1	GC	0	0
38	7	GC	0	0
39	7	GG	1	1
40	5	CC	0	0
41	7	GC	1	1
42	4	GG	1	3
43	8	GC	1	1
44	2	GC	0	0
45	3	GC	1	3
46	1	GG	0	0
47	3	GG	1	4

Patients	Age (years)	rs2736191	Mild Malaria	Number of mild malaria episodes
48	8	GG	1	3
49	1	GG	0	0
50	5	GG	0	0
51	6	GG	0	0
52	8	GG	1	1
53	5	GG	0	0
54	1	GC	0	0
55	5	GC	1	1
56	1	GG	0	0
57	2	GG	0	0
58	5	GG	0	0
59	9	GG	0	0
60	1	CC	1	1
61	4	GC	0	0
62	9	GG	1	1
63	1	CC	0	0
64	1	GG	0	0
65	8	GG	1	1
66	4	GG	0	0
67	8	GG	1	1
68	3	GG	1	1
69	6	GG	1	1
70	8	GG	0	0
71	9	GG	0	0
72	9	GC	0	0
73	1	GG	1	1
74	6	CC	1	3
75	4	GC	0	0
76	1	GC	0	0
77	4	GG	1	1
78	6	GC	0	0
79	5	GC	0	0
80	8	GG	1	3
81	3	GG	1	4
82	2	GG	0	0
83	2	GC	1	2
84	2	GG	0	0
85	7	GC	1	1
86	7	GG	0	0
87	8	GC	1	1
88	1	GC	0	0
89	8	GG	0	0
90	9	GG	0	0
91	4	GC	1	2
92	7	GG	1	1
93	2	GG	0	0
94	6	GG	1	4
95	4	GC	1	1

Patients	Age (years)	rs2736191	Mild Malaria	Number of mild malaria episodes
96	6	GG	1	1
97	9	GC	0	0
98	1	GC	1	2
99	1	GG	0	0
100	1	CC	0	0
101	8	GC	1	2
102	7	GC	1	2
103	4	GG	1	3
104	1	GG	0	0
105	2	GC	1	1
106	5	GG	1	2
107	2	GG	0	0
108	2	GC	0	0
109	8	GC	0	0
110	2	GC	0	0
111	4	GG	1	1
112	6	GC	1	1
113	2	GG	0	0
114	2	GG	1	1
115	2	GG	1	1
116	7	GC	1	2
117	5	GG	1	1
118	3	GC	0	0
119	2	GC	1	1
120	1	GC	0	0
121	6	GG	1	1
122	2	GG	0	0
123	1	GC	1	1
124	5	GC	1	2
125	5	GG	0	0
126	3	GC	0	0
127	7	GC	1	1
128	4	GC	0	0
129	5	GG	1	2
130	1	GG	1	1
131	6	GC	1	4
132	2	GC	1	1
133	7	GG	1	1
134	1	GC	1	3
135	2	GC	0	0
136	6	GG	0	0
137	3	GC	1	2
138	8	GG	1	4
139	3	GG	0	0
140	2	GC	1	1
141	5	GC	1	4
142	1	GG	0	0
143	3	GG	1	3

Patients	Age (years)	rs2736191	Mild Malaria	Number of mild malaria episodes
144	5	GG	0	0
145	7	GC	1	2
146	2	GG	1	1
147	2	CC	0	0
148	2	GG	0	0
149	6	GG	0	0
150	3	GC	0	0
151	2	GC	0	0
152	2	CC	1	3
153	2	CC	0	0
154	5	GG	1	1
155	2	GC	1	1
156	5	GG	1	1
157	6	GC	0	0
158	2	CC	0	0
159	9	GC	0	0
160	1	GC	1	2
161	3	GG	1	1
162	8	GC	1	2
163	1	GC	1	1
164	6	GG	1	1
165	2	GC	1	3
166	3	GG	1	1
167	6	GG	1	1
168	8	GC	1	1
169	8	GC	1	1
170	6	GC	1	2
171	5	GG	0	0
172	3	GG	0	0
173	2	GG	1	4
174	3	GG	0	0
175	7	GC	0	0
176	1	GC	1	1
177	9	GG	1	1
178	9	GC	1	2
179	5	GC	1	1
180	4	GC	1	1
181	5	GG	1	2
182	6	GC	1	1
183	2	GG	1	1
184	3	GG	0	0
185	1	GC	0	0
186	4	GC	1	1
187	2	GG	1	5
188	2	GG	1	1
189	3	GG	1	1
190	1	GG	0	0
191	1	CC	0	0

Patients	Age (years)	rs2736191	Mild Malaria	Number of mild malaria episodes
192	4	GC	1	1
193	6	GC	1	2
194	1	GC	1	1
195	3	GG	1	1
196	4	GG	1	2
197	1	GG	1	2
198	1	GG	1	1
199	7	GC	1	3
200	9	GG	1	1
201	4	GG	0	0
202	6	GG	0	0
203	4	GC	1	1
204	5	GC	1	1
205	9	GC	1	1
206	6	CC	1	3
207	3	GG	0	0
208	7	GC	0	0
209	3	GG	0	0
210	6	GG	0	0
211	8	CC	1	4
212	3	GG	1	1
213	1	GC	0	0
214	1	GG	0	0
215	7	GG	0	0
216	3	GC	0	0
217	8	CC	0	0
218	3	GG	0	0
219	2	GG	1	1
220	3	GG	0	0
221	9	GG	1	2
222	6	GC	1	2
223	2	GG	1	1
224	5	GG	0	0
225	2	GG	0	0
226	1	GG	0	0
227	7	GG	0	0
228	1	GG	0	0
229	8	GG	1	2
230	9	GG	0	0
231	6	GG	0	0
232	1	GC	0	0
233	2	GG	0	0
234	4	GG	1	2
235	6	GG	0	0
236	4	CC	1	1
237	3	GG	1	1
238	7	GG	0	0
239	2	CC	0	0

Patients	Age (years)	rs2736191	Mild Malaria	Number of mild malaria episodes
240	6	GG	0	0
241	2	GC	1	2
242	2	GG	1	1
243	7	GG	0	0
244	4	GC	0	0
245	1	GG	0	0
246	7	GG	1	2
247	5	CC	1	1
248	7	CC	1	3
249	7	GC	1	4
250	8	GG	0	0
251	2	GG	1	1
252	7	GC	0	0
253	3	GC	0	0
254	5	GC	1	1
255	2	GC	0	0
256	5	GC	0	0
257	8	GC	1	1
258	5	GG	0	0
259	1	GG	1	1
260	6	GC	1	3
261	8	GC	1	1
262	6	GC	0	0
263	3	GC	1	3
264	1	CC	1	1
265	5	GG	0	0
266	8	GG	1	4
267	1	GC	1	1
268	8	GG	1	1
269	4	GG	0	0
270	4	GG	0	0
271	2	GG	1	1
272	1	CC	0	0
273	8	GG	0	0
274	5	GG	0	0
275	1	GG	0	0
276	4	CC	1	1
277	2	GC	1	1
278	5	GG	1	2
279	8	GG	1	2
280	5	GC	1	1
281	8	GC	1	1
282	2	GG	1	2
283	1	GG	0	0
284	5	GC	1	1
285	1	GG	1	2
286	8	GC	1	2
287	7	GC	1	2

Patients	Age (years)	rs2736191	Mild Malaria	Number of mild malaria episodes
288	5	GC	0	0
289	3	GC	1	1
290	1	GC	0	0
291	4	GG	0	0
292	9	GG	0	0
293	8	GC	1	1
294	1	GG	1	1
295	2	GC	0	0
296	3	GC	1	1
297	2	GG	0	0
298	5	GG	1	1
299	1	GG	0	0
300	2	CC	0	0
301	3	GG	0	0
302	2	GG	0	0
303	2	GG	1	1
304	3	GG	0	0
305	5	GG	1	1
306	1	GG	0	0
307	2	GG	1	1
308	1	GC	0	0
309	3	GC	1	1
310	1	CC	0	0
311	6	GG	1	1
312	1	GG	0	0
313	6	GG	1	2
314	9	GC	1	1
315	1	CC	1	1
