# Supplementary material for: Beyond genome-wide scan: Association of a cis-regulatory NCR3 variant with mild malaria in a population living in the Republic of Congo
Source: PLoS One. 2017 Nov 9;12(11):e0187818. doi: 10.1371/journal.pone.0187818 (PMC5679660; doi:10.1371/journal.pone.0187818)
Supplement: S2 Table — (PDF) [file pone.0187818.s002.pdf]

Table S2. Proportion of mild malaria episodes according to rs2736191 polymorphism in children < 5 years old

Genotype	Number of mild malaria episodes						Total
	0	1	2	3	4	5	
GG	46 (54.8%)	29 (50%)	6 (46.2%)	4 (44.4%)	3 (75%)	1 (100%)	89 (52.7%)
GC	28 (33.3%)	23 (39.7%)	7 (53.8%)	4 (44.4%)	1 (25.0%)	0 (0%)	63 (37.3%)
CC	10 (11.9%)	6 (10.3%)	0 (0%)	1 (11.1%)	0 (0%)	0 (0%)	17 (10.1%)
Total	84 (100%)	58 (100%)	13 (100%)	9 (100%)	4 (100%)	1 (100%)	169 (100%)
