# Supplementary material for: Beyond genome-wide scan: Association of a cis-regulatory NCR3 variant with mild malaria in a population living in the Republic of Congo
Source: PLoS One. 2017 Nov 9;12(11):e0187818. doi: 10.1371/journal.pone.0187818 (PMC5679660; doi:10.1371/journal.pone.0187818)
Supplement: S3 Table — (PDF) [file pone.0187818.s003.pdf]

Table S3. Proportion of mild malaria episodes according to rs2736191 polymorphism in children ≥ 5 years old

Genotype	Number of mild malaria episodes						Total
	0	1	2	3	4	5	
GG	37 (68.5%)	25 (49%)	10 (41.7%)	2 (28.6%)	4 (44.4%)	1 (100%)	79 (54.1%)
GC	15 (27.8%)	24 (47.1%)	14 (58.3%)	2 (28.6%)	3 (33.3%)	0 (0%)	58 (39.7%)
CC	2 (3.7%)	2 (3.9%)	0(0%)	3 (42.9%)	2 (22.2%)	0 (0%)	9 (6.2%)
Total	54 (100%)	51 (100%)	24 (100%)	7 (100%)	9 (100%)	1 (100%)	146 (100%)
